# Are ADHD trajectories shaped by the social environment? A longitudinal study of maternal influences  on the preschool origins of delay aversion

**DOI:** 10.1111/jcpp.14103

**Published:** 2024-12-22

**Authors:** Wendy W.Y. Chan, Kathy Kar‐man Shum, Johnny Downs, Edmund J.S. Sonuga‐Barke

**Affiliations:** ^1^ School of Academic Psychiatry, Institute of Psychiatry Psychology & Neuroscience, King's College London London UK; ^2^ Department of Psychology The University of Hong Kong Hong Kong China; ^3^ Department of Child & Adolescent Psychiatry Aarhus University Aarhus Denmark

**Keywords:** ADHD, development, delay aversion, preschoolers, parenting, social factors

## Abstract

**Background:**

Attention‐deficit/hyperactivity disorder (ADHD) is commonly attributed to neuro‐cognitive deficits of genetic and/or prenatal/perinatal environmental origins. Sonuga‐Barke proposed an alternative formulation, suggesting that ADHD behaviors are functional expressions of delay aversion—a strong motivational disposition to avoid or escape negative affective states evoked by delay. It is hypothesized that the strength of this disposition, though neuro‐biologically rooted, is exacerbated by early negative social interactions during waiting‐related encounters. This paper reports findings from an initial proof‐of‐concept study that specifically tests this hypothesis in a nonclinical sample.

**Methods:**

Preschoolers (*n* = 112; mean age = 46.2 months) and their parents from London, UK, and Hong Kong participated in a longitudinal study. The Parent–Child Delay Frustration Task (PC‐DeFT) and two nonwaiting control tasks were administered at baseline. Children's performance, behavioral and emotional responses, and parents' reactions were observed. Teachers rated children's ADHD behaviors and delay aversion at baseline and follow‐up (12–18 months later).

**Results:**

At baseline, children's maladaptive performance and parental negative reactions during the PC‐DeFT were correlated with each other and with teacher ratings of ADHD and delay aversion. Negative parental reactions during the PC‐DeFT at baseline predicted an increase in teacher‐rated ADHD behaviors at follow‐up, but similar associations were not observed for baseline parental responses in the nonwaiting tasks. The increase in child ADHD symptoms associated with negative parental reactions at baseline was statistically mediated by delay aversion. These longitudinal effects were consistent across the UK and HK samples.

**Conclusions:**

The findings provide the first evidence that parent's negative reactions to preschooler's attempts to manage delay are associated with increases in ADHD behaviors overtime, and linked to delay aversion increases. They underscore the potential significance of the early social environment as a contributor to developmental trajectory of ADHD behaviors. Future studies with clinical samples over an extended time‐frame using a range of different aversive environments (i.e. difficult tasks to complete) are indicated.

## Introduction

Attention‐deficit/hyperactivity disorder (ADHD) is a neurodevelopmental condition characterized by age‐inappropriate and pervasive patterns of hyperactivity, inattentive and impulsive behaviors, which cause impairment across functional domains (American Psychiatric Association, 2022; Posner, Polanczyk, & Sonuga‐Barke, 2020). Existing data is consistent with models that see ADHD as the result of subtle alterations in multiple brain systems creating deficits in higher order cognition – these being the result of the interaction between genetic and/or prenatal or perinatal environmental origins (Sonuga‐Barke et al., 2023). These models consider the developmental course of ADHD behaviors as largely impervious to common postnatal social environmental influences (e.g. normal variations in parenting), although there is evidence for a role of extreme institutional deprivation (Kennedy et al., 2016).

An alternative sociomotivational account of the development of ADHD behaviors was proposed by Sonuga‐Barke based on functional analysis (Sonuga‐Barke, 1994, 2005; Sonuga‐Barke, Wiersema, van der Meere, & Roeyers, 2010). The delay aversion hypothesis postulates that ADHD symptoms are functional expressions of delay aversion—a negative affective response to delay, manifest as attempts to avoid or reduce waiting experiences. More specifically, the hypothesis proposes an explanation for each of the cardinal ADHD symptoms—hyperactivity, impulsivity and inattention. It hypothesizes that delay aversion presents as impulsiveness in situations where delay can be escaped (e.g. leading to fast and inaccurate responding). While in contexts where delay is fixed and unavoidable, it presents as inattention and hyperactivity. This latter notion is based on extensive evidence that alterations to patterns of attention and activity may create stimulation that can change the way that time is experienced by an individual (e.g. Zakay, 1992; Zakay & Block, 1997; Zakay & Tsal, 1989). This in turn led to the insight that the negative affect associated with waiting could be reduced by increasing activity or refocusing attention to speed up the passage of time (e.g. Sonuga‐Barke, 1994). The delay aversion model views ADHD behaviors as context‐dependent, suggesting that the expression of underlying motivational tendencies varies in intensity based on the level of delay present in a given situation.

Support for the delay aversion hypothesis comes from experimental and neuroimaging studies. Research has consistently shown greater impulsivity in individuals with ADHD when given the opportunity to escape delay (Dalen, Sonuga‐Barke, Hall, & Remington, 2004; Marco et al., 2009; Sonuga‐Barke, Houlberg, & Hall, 1994; Sonuga‐Barke, Taylor, Sembi, & Smith, 1992) and increased levels of ADHD‐related behavioral agitation and distraction during extended periods of fixed delays (Mies et al., 2018). The latter effect increases as a function of delay length (Bitsakou, Antrop, Wiersema, & Sonuga‐Barke, 2006; Marx, Hacker, Yu, Cortese, & Sonuga‐Barke, 2021). Experimental studies have shown that children with ADHD exhibit significantly higher levels of physical activity and inattention, such as running around or touching objects, in unstimulating waiting situations compared to controls. However, when extra stimulation is added, group differences in activity and attention are reduced (Antrop et al., 2006; Antrop, Roeyers, Van Oost, & Buysse, 2000). Perhaps the most compelling evidence linking ADHD to delay aversion comes from brain imaging studies showing that inescapable delay triggers a specific hyperactivation of brain systems known to be activated during aversive events (e.g. amygdala), and the strength of activation being proportionate to the length of the delay imposed (Van Dessel et al., 2018).

Later formulations of the model incorporated an account of the genesis of delay aversion and its role in influencing the developmental course of ADHD behaviors (Marco et al., 2009; Sonuga‐Barke, 2005). Specifically, it is hypothesized that the strength of the delay aversion disposition, though neuro‐biologically rooted, is exacerbated by negative early social interactions with significant others (e.g. parents) during waiting‐related encounters—effects evoked and maintained by maladaptive behaviors on the part of both the child (e.g. failure to wait, loss of concentration, acting up when bored) and adult (e.g. criticism, intrusiveness). As a result, delay acquires a more negative significance, which creates a greater delay escape‐motivation. This in turn is proposed to lead to a subsequent increase in ADHD behaviors—as the child acts to escape the aversive experience of delay (Cartwright et al., 2011; Sonuga‐Barke, Auerbach, Campbell, Daley, & Thompson, 2005).

This hypothesis of the origins of delay aversion and its role in shaping the developmental course of ADHD behaviors has not been tested. There is evidence that parents of children with ADHD exhibit more reactive, punitive, and controlling behaviors in their general interaction with their children (Johnston & Mash, 2001; Modesto‐Lowe, Danforth, & Brooks, 2008; Teixeira, Marino, & Carreiro, 2015). Longitudinal and experimental studies have suggested that this association is largely driven by evocative child‐effects (Burt, McGue, Krueger, & Iacono, 2005; Lifford, Harold, & Thapar, 2008; Schachar, Taylor, Wieselberg, Thorley, & Rutter, 1987; Shelleby & Ogg, 2020). Evidence that such negative parental responses increase the risk of subsequent ADHD is lacking (Lifford, Harold, & Thapar, 2009; Tarver, Daley, & Sayal, 2014), with their effects being most clearly seen in an increase of conduct problems (Chronis et al., 2007; Daley et al., 2014; Pfiffner, McBurnett, Rathouz, & Judice, 2005). However, studies have not examined the differential impact of delay‐context (i.e. waiting vs. nonwaiting) on the longitudinal relationships between parent–child interaction and subsequent ADHD—the key test of the proposed account.

Here we report an initial proof‐of‐concept longitudinal study to test, in a nonclinical sample, whether parental reactions to their preschool children's responses during waiting (as opposed to nonwaiting settings) at baseline increase their children's ADHD behaviors at follow‐up (12–18 months later) and whether these effects are mediated by the emergence of delay aversion. We developed a new experimental task—the Parent–Child Delay Frustration Task (PC‐DeFT) – to examine parents' reactions to their children's behaviors when they had to wait unexpectedly for an unspecified duration. In this task, parents and children played a simple and enjoyable shopping game where the presentation of a *Go*‐signal was sporadically and unpredictably interrupted with enforced periods of waiting of different delay lengths. The children's emotional and behavioral responses to these delay periods and their parent's reactions were coded. Our sample included families from two cultures with different attitudes to parenting and children's behavior (the UK and Hong Kong). We did this to broaden the range of likely parental reactions. In particular, HK parents, compared to Western counterparts, have been found to exhibit stricter standards with regard to children's behavior and are more directive and controlling (Chan, Shum, & Sonuga‐Barke, 2022; Chao, 1994; Chen, 2005; Lam & Ho, 2010; Thompson et al., 2017).

In this study, we explored several key questions regarding ADHD behaviors and parental associates. First, we examined whether children's ADHD behaviors were correlated with their waiting‐related responses observed during the PC‐DeFT at baseline. Second, we also investigated if these responses were related to parental negative reactions during waiting. Third, we assessed whether parental negative reactions at baseline predicted ADHD behaviors at follow‐up (12–18 months later) and if this association persisted after controlling for children's baseline ADHD behaviors and waiting‐related responses. Fourth, where a link between baseline parental reactions and later ADHD was found, we evaluated whether changes in children's delay aversion levels statistically mediated this relationship. Fifth, we further explored whether similar relationships between delay aversion, ADHD, and parental reactions existed in nonwaiting tasks. Finally, we examined whether these associations were consistent across both Hong Kong and UK settings.

We hypothesized that:

1. Children's baseline ADHD behaviors would be correlated with their maladaptive waiting‐related responses during the PC‐DeFT, and both would be associated with parental negative reactions during waiting.
2. In keeping with our overall hypothesis, parental negative reactions during waiting at baseline would predict children's ADHD behaviors at follow‐up, and this relationship would be mediated by changes in children's levels of delay aversion.
3. Parental reactions in nonwaiting settings at baseline would not predict ADHD behaviors at follow‐up after controlling for the baseline ADHD behaviors and waiting‐related responses.
4. While HK parents would be more reactive during waiting than UK parents, the association between parental reactions, delay aversion and ADHD symptom changes would be invariant across the HK and UK samples.

## Methods

This work was funded by the Centre for Doctoral Studies at King's College London and received ethical approval from the Research Ethics Committees at King's College London (KCL; reference: HR‐18/19‐8506) and the University of Hong Kong (HKU; reference: EA1812027) in 2019.

### Participants

To investigate early ADHD and delay aversion trajectories, this study focused on preschoolers, recognizing that ADHD behaviors emerging in early years persist and have comparable impact to those in school‐aged children (Biederman, Petty, Evans, Small, & Faraone, 2010; Fantuzzo et al., 2001; Sonuga‐Barke, Dalen, & Remington, 2003; Sonuga‐Barke, Thompson, Stevenson, & Viney, 1997). Participants were recruited via local nurseries, preschools and online parent groups using social media adverts in London, UK, and HK. At the initial screening stage, after being informed about the nature of the study and their right to participate and withdraw voluntarily, 189 preschool children and their parents agreed to participate in the screening and signed the informed consent form (UK: *n* = 68, 51% male; HK: *n* = 121, 58% male).

The screening questionnaires completed by teachers and parents gathered basic demographic information about the child participants, including whether the child had a diagnosis of special educational needs and/or pervasive developmental disorders (e.g. autism spectrum disorder). It also asked about the primary language spoken at home and at school. Thirty children (*n* ^UK^ = 13 and *n* ^HK^ = 17) were excluded based on the following criteria: outside the age range; existing diagnosis; age‐inappropriate level of comprehension abilities in spoken English (UK) or Cantonese (HK); teacher nonengagement; family unable to attend testing sessions. No participants had a formal ADHD diagnosis or were taking ADHD medications. To ensure we included child participants with a range of levels of activity and attention problems and compared like with like across cultures, children were screened for their ADHD behaviors using the five‐item hyperactivity/inattention subscale of the Strengths and Difficulties Questionnaire completed by parents and teachers (SDQ, version T2‐4). We oversampled participants and then excluded 47 children to balance the degree of ADHD behaviors at a group level in HK and UK samples. In the final sample, most participants (*n* = 69, 61.6%) had low or average levels of ADHD symptoms (subscale score ≤ 4). Around one‐third (*n* = 33, 33.0%) showed slightly elevated symptoms (subscale score = 5–6), while a small number (*n* = 6, 5.4%) exhibited high levels of symptoms (subscale score ≥ 7). The number of children having elevated levels of ADHD symptoms rated by parents or teachers (subscale score ≥ 5) in the final UK and HK samples was not statistically different, *χ* ^2^(1) = 1.27, *p* = .26. The average SDQ subscale scores in the UK (*M* = 3.40, *SD* = 2.34) and HK sample (*M* = 3.67, *SD* = 1.79) were not statistically different, *F*(1, 110) = 2.33, *p* = .130. Full baseline data were available for 112 parent–child dyads (*n* ^UK^ = 55 and *n* ^HK^ = 57). All parent participants in this sample were mothers, while the percentage of boys in the child sample was 56% (*n* = 63).

Families were contacted 11 months after their baseline testing session to offer participation in the follow‐up assessment; 79.5% of them completed the follow‐up (*n* ^UK^ = 39 and *n* ^HK^ = 50; females = 42 and males = 47). Reasons for drop‐out included moving abroad, time constraint and being uncontactable. The mean age of child participants at baseline and follow‐up was 46.20 months (*SD* = 5.73; range = 36.92–59.24) and 60.85 months (*SD* = 7.76; range = 48.36–81.30), respectively. The mean IQ of participants was 106.95 (*SD* = 11.53; range = 82–132, i.e. all children met the inclusion criteria of IQ ≥ 80).

### Procedures

At baseline parent–child dyads were invited to attend in‐person testing sessions which took place in quiet rooms either at King's College London or the University of Hong Kong. The testing was conducted by trained experimenters (one in each university). Participants were briefed that this was a longitudinal cross‐cultural study exploring preschoolers' behaviors in tasks that require patience and waiting. After giving their informed consent, the dyads completed 8 min of free play. Parents were then asked to instruct their children to tidy up their toys without assistance. After that, the dyads completed the PC‐DeFT waiting task. The research team presented a certificate and book voucher to each participating dyad as a token of appreciation. Parents and the child's class teachers completed sets of questionnaires at baseline (2019) and again at follow‐up 12–18 months later (2020/21). The average time difference between baseline and follow‐up assessment was 14 months ($\bar{x}$ ^HK^ = 12 months; $\bar{x}$ ^UK^ = 18 months).

### Measures

#### Screening measures

ADHD symptom screener: The parent and teacher versions of the Strengths and Difficulties Questionnaire (SDQ, version T2‐4) are widely used psychometrically strong, brief screening questionnaire designed for research/clinical purposes (Goodman, 1997). The hyperactivity/inattention subscale consists of five items: two measuring inattention, two hyperactivity and one impulsivity. The original English language version was used in the UK. A validated Chinese translation was employed in HK (Lai et al., 2010).

Intelligence: Child IQ was estimated using the Block Design and Vocabulary subtests of Wechsler Preschool and Primary Scale of Intelligence (WPPSI‐III; Wechsler, 2003). The WPPSI measures the cognitive ability of preschoolers and young children between 2 years 6 months and 7 years and 3 months. The English (UK) and Traditional Chinese language versions were used in the UK and HK, respectively.

#### Parent–child interaction tasks

We measured parents' reactions to their children's responses in three settings: one incorporating unexpected delay of different duration on some trials (*PC‐DeFT*), one with no delay but the need to comply with parent's request (clean‐up) and one with no delay and no planned request (free play).

**Figure 1:**
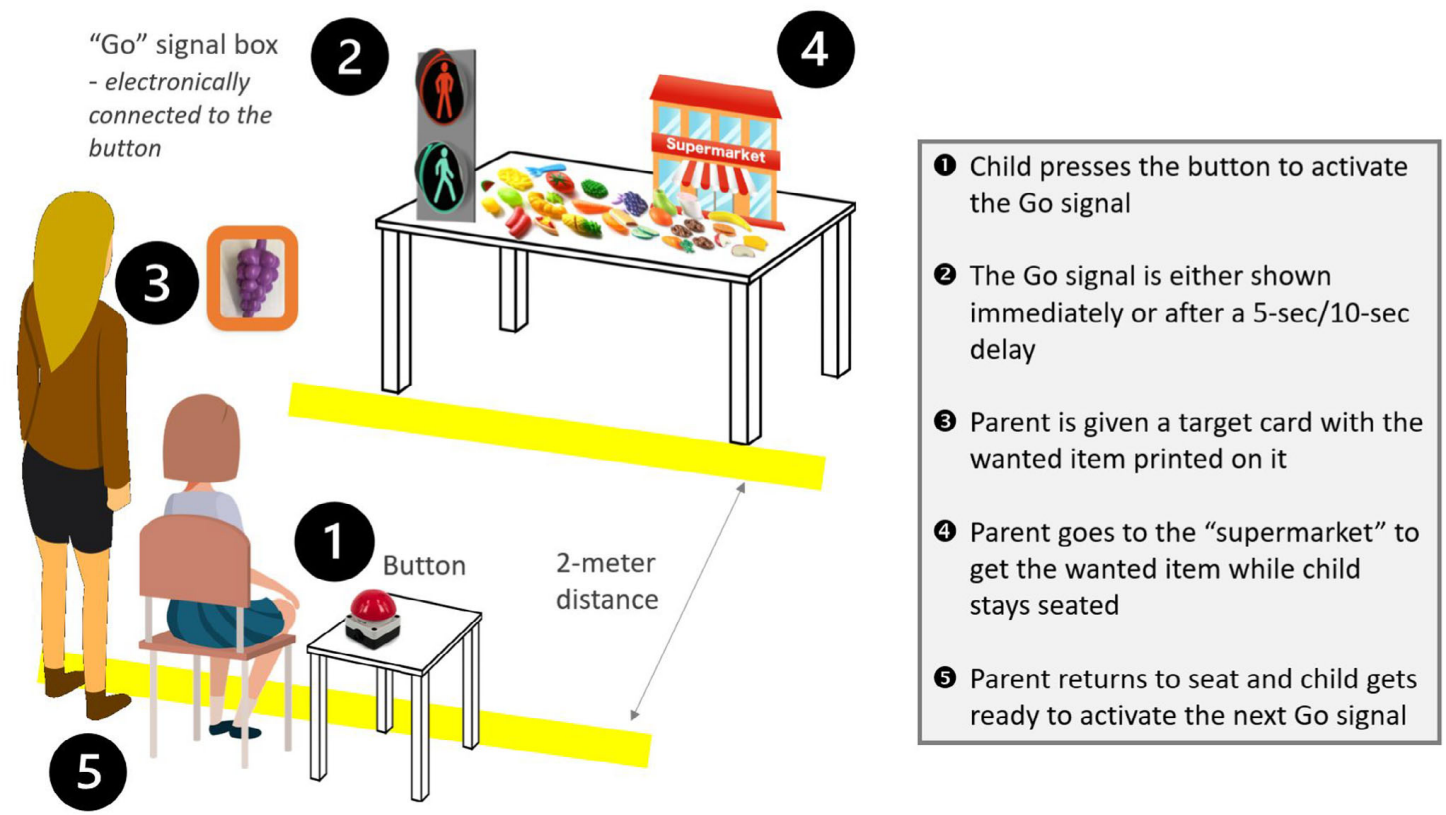
The set up and procedures of the Parent–Child Delay Frustration Task (PC‐DeFT) In the PC‐DeFT, participants were shown a pedestrian traffic light system, then in each trial, the child participants had to (1) press the button and (2) when the light turned green (immediately or after a short period of delay), the parent participants would be (3) shown a shopping card. After that, parents could (4) go to the supermarket to pick up the toy while the children have to stay seated. When the parents (5) returned to their seat, the children can press the button again

Parent–Child Delay Frustration Task: The PC‐DeFT was developed specifically for this study. It is a computerized task similar to the Preschool Delay Frustration Task (P‐DeFT; Chan, Shum, Downs, & Sonuga‐Barke, 2024). Modifications were made with regard to the role of parents and children. The original P‐DeFT was designed as a simple and enjoyable ‘shopping’ game. The set up includes a traffic light system shown on a screen and a ‘crossing’ button which can be pressed to change the red *Wait* signal to a green *Go‐*signal. In each trial, participants are first shown the *Wait* signal. They then have to press the button to elicit the *Go‐*signal. In the P‐DeFT, the child participant can then visit the ‘toy supermarket’ to get the target object as shown by the experimenter when they see the *Go‐*signal. In this modified PC‐DeFT version, the dyads are told to work together to complete the task—child participants are in charge of the button pressing while the parents are responsible for getting the target object from the ‘supermarket’ when the red signal turns green. Children are reminded to stay at their seat all the time (as illustrated in Figure 1). There is a total of 18 PC‐DeFT trials. During the majority of trials (*n* = 12), the *Go‐signal* is shown immediately after the child presses the crossing button (i.e. no *pre‐Go‐signal* delay). In the remaining six trials, a *pre‐Go‐signal waiting period* (either 5‐s or 10‐s; three trials each) is imposed in a pseudo‐random order. Participants are not informed beforehand about the presence of these extra delay periods.

In this task, four measures are used to assess the children's delay‐related responses: (a) number of button presses per second during the *pre‐Go‐signal* waiting period which reflects their intention to stop the wait; (b) activity level tracked using an unobtrusive wrist‐worn triaxial actigraph unit accelerometer; (c) observed delay‐related behavioral agitation (i.e. squirming/fidgeting) and (d) negative emotional responses (i.e. observed frustration) which are coded using a 4‐point scale with 0 = *None/ Very rare*, 1 = *A little*; 2 = *Quite a lot* and 3 = *A lot*. Four coders were trained using videos collected from the pilot to a 90% consensus level prior to the official video coding. Inter‐rater reliability for the behavioral and affective codes was excellent—with ICC estimates of .98 and .95, respectively, indicating an excellent agreement between the raters.

The parents' reactions to their children were observed and coded using an adaptation of the Parent–Child Interaction System (PARCHISY; Deater‐Deckard, 2000; Deater‐Deckard, Pylas, & Petrill, 1997). This coding system had been used extensively across cultures in research with both typically developing population and children with externalizing or internalizing behavior problems (Aspland & Gardner, 2003; Funamoto & Rinaldi, 2015). Codes used in the current study (see Supporting Information for details) included both positive and negative reactions to their child's behavior: (a) positive content, (b) positive affect, (c) reciprocity, (d) negative content and (e) negative affect. Researchers rated the parents' reactions on a 7‐point scale from 1 = *None or never* to 7 = *Always or constantly*.

Free play and clean‐up: Unstructured free play lasted 8 min, during which the dyads were allowed to choose any activity or toys available and play together as they would in everyday life. After the eight‐minute play session, the parents were asked to request their children to tidy up all the toys but were reminded not to give any actual assistance. There was no time limit for completing the tidy up; experimenters recorded the exact start and end time. The time used to complete clean‐up ranged between 21 s and 6 min 12 s. Parental reactions were coded in the same way as for the PC‐DeFT. In this sample, the average ICC estimates for the five codes in free play, clean‐up and PC‐DeFT were .96 (range = .90–1.00), .80 (range = .68–.88) and .86 (range = .82–.93), respectively, indicating good‐to‐excellent inter‐rater agreement.

#### Teacher ratings

We used teachers' ratings of children's ADHD behaviors and delay aversion rather than parents' ratings as both baseline and outcome variables at follow‐up assessments to ensure independence of rating source and avoid shared‐method variance.

ADHD symptom rating: Children's hyperactivity/inattention behaviors at baseline were rated by teachers on the Strengths and Difficulties Questionnaire (SDQ, version T2‐4; Goodman, 1997; Lai et al., 2010). At follow‐up, teachers rated the children's frequency of occurrence of the 18 DSM‐IV ADHD behaviors using the ADHD Rating Scale IV (DuPaul, Power, Anastopoulos, & Reid, 1998) adapted for preschoolers with a 4‐point scale: 0 = *Never or rarely*, 1 = *Sometimes*, 2 = *Often* and 3 = *Very often* (ADHD‐RS‐IV‐P; McGoey et al., 2007). A total score was computed by adding the scores of all the items. The ADHD Rating Scale IV had been validated in a large sample of 1,616 Chinese children (Su et al., 2015); the psychometric properties of the translated Chinese ADHD Rating Scale IV have been found to be comparable to the original English version, with high internal consistency, good test–retest reliability, and high convergent and discriminant validity. In this sample, Cronbach's *α*s for the full scale at baseline and follow‐up were .91 and .93, respectively; test–retest reliability was .84.

Delay aversion rating: The Quick Delay Questionnaire (QDQ) was originally designed to measure adults' self‐reported delay‐related behaviors (Clare, Helps, & Sonuga‐Barke, 2010). Markomichali (2015) adapted it to be used for preschoolers as rated by teachers or parents. There are 10 items in total tapping two aspects: (i) Delay Aversion (DA, e.g. ‘Hates waiting for things’) and (ii) Delay Discounting (DD; e.g. ‘Often gives up on things he or she can't have immediately’). At follow‐up, teachers rated children' on a 5‐point Likert scale from 1 = *Not at all like him/her* to 5 = *Very much like him/her*. In this sample, Cronbach's *α* for the 10 items was .84 and test–retest reliability was .75.

### Data analysis

#### Preparatory analyses

A small proportion of the PC‐DeFT data (5%) and actometer reading (4%) were missing due to technical issue (e.g. program crashing, data storage error in actometer). Where data were missing, we used pairwise deletion to optimize data availability. To minimize the need for multiple testing, we reduced the number of variables by running two‐factor analyses to explore the relationships between the: (i) four measures of children's delay‐related responses and (ii) the five indices of parents' reactions with their children's delay‐related behaviors. Factor scores were calculated and used in the subsequent analyses. We then explored which demographic and background factors should be included in our models as covariates. We did this by examining which of these were associated with our main outcomes and mediators ‐ ADHD behaviors and delay aversion at follow‐up.

Research questions 1 and 2: We first conducted baseline correlation analyses to examine the relationship between children's ADHD behaviors and maladaptive waiting‐related responding and the relationship between these and parental reactions during PC‐DeFT.

Research questions 3, 4 and 5: We then explored the association between parental negative reactions in waiting/nonwaiting settings at baseline and children's ADHD behaviors at follow‐up using correlational and regression analyses, controlling for children's baseline ADHD behaviors and maladaptive waiting‐related responses. Regression analyses looked at the relative predictive power of parental reactions in waiting and nonwaiting settings. To explore if these relationships were mediated by children's delay aversion, sets of PROCESS macro test of mediation (model 4; 5,000 bootstrap samples) were run with parental responses during waiting and nonwaiting settings as predictors, while controlling for children's waiting‐related performance and ADHD symptom levels at baseline.

Research question 6: We ran ANOVAs to explore whether there were any significant differences between UK and HK participants on their waiting‐related responses and reactions and ratings on delay aversion and ADHD behaviors. We then tested if national group moderated the relationship between parental responses during waiting and ADHD behaviors at follow‐up via the children's delay aversion ratings by first running spilt‐sample correlation analyses, then a PROCESS macro test of moderated mediation (model 59, 5,000 bootstrap samples).

## Results

### Descriptive statistics

Table 1 presents the demographic characteristics of the UK and HK participants at both baseline and follow‐up. The two samples did not differ significantly in most aspects. UK and HK children's age at baseline was not statistically different, but the UK children's average age at follow‐up when their parents and teachers completed the questionnaire (*M* = 65.1, *SD* = 8.8) was greater than that of HK children (*M* = 57.6, *SD* = 4.8), *F*(1, 87) = 26.37, *p* < .001. During the pandemic, contacting parents and teachers was challenging and a large number of parents and teachers, especially those in the UK, needed additional time to complete the questionnaires due to the extra childcare and online teaching support in that period.

**Table 1:** Demographic characteristics of participants in UK and HK at baseline (T1) and follow‐up (T2)

	T1		T2		Statistical comparison	
	UK (*n* = 55)	HK (*n* = 57)	UK (*n* = 39)	HK (*n* = 50)	T1	T2
Child characteristics						
Age (months) – mean (*SD*)	46.55 (6.49)	45.86 (4.91)	65.06 (8.79)	57.56 (4.80)	*F*(1, 110) = 0.41, *p* = .526	*F*(1, 87) = 26.37, *p* < .001
Female – *n* (%)	25 (45.45)	24 (42.11)	19 (48.72)	23 (46.00)	*χ* ^2^(1) = 0.13, *p* = .721	*χ* ^2^(1) = 0.07, *p* = .799
IQ – mean (*SD*)	108.72 (12.20)	105.26 (10.69)	/	/	*F*(1, 109) = 2.53, *p* = .114	/
Parent characteristics *n* (%)						
Full‐time employment	18 (32.73)	27 (47.37)	12 (30.77)	24 (48.00)	*χ* ^2^(1) = 2.50, *p* = .114	*χ* ^2^(1) = 2.70, *p* = .100
Age group						
25–34	11 (20.00)	22 (38.60)	4 (10.26)	11 (22.00)	*χ* ^2^(2) = 4.71, *p* = .095	*χ* ^2^(2) = 2.90, *p* = .235
35–39	30 (54.55)	23 (40.35)	24 (61.54)	23 (46.00)		
Over 40	14 (25.45)	12 (21.05)	11 (28.21)	16 (32.00)		
Highest education level						
Secondary	1 (1.82)	15 (26.32)	0 (0)	13 (26.00)	*χ* ^2^(3) = 26.07, *p* < .001	*χ* ^2^(3) = 27.27, *p* < .001
Higher education	3 (5.45)	11 (19.30)	2 (5.13)	9 (18.00)		
Bachelor	22 (40.00)	21 (36.84)	12 (30.77)	20 (40.00)		
Master or above	29 (52.73)	10 (17.54)	25 (64.10)	8 (16.00)		
Monthly household income						
Below £2,000	4 (7.27)	6 (10.53)	2 (5.13)	9 (18.00)	*χ* ^2^(3) = 5.33, *p* = .149	*χ* ^2^(3) = 5.28, *p* = .152
£2,000–2,999	1 (1.82)	7 (12.28)	2 (5.13)	6 (12.00)		
£3,000–3,999	8 (14.55)	8 (14.04)	5 (12.82)	6 (12.00)		
Above £4,000	42 (76.36)	36 (63.16)	30 (76.92)	29 (58.00)		
Ethnicity						
White	40 (72.73)	0 (0)	31 (79.49)	0 (0)	Not applicable	
Asian	8 (14.55)	57 (100.00)	7 (17.95)	50 (100.00)		
Black/mixed	7 (12.73)	0 (0)	1 (2.56)	0 (0)		

### Data reduction

Table 2 shows the intercorrelations between child's waiting‐related responses and parent's reactions to child's behaviors during PC‐DeFT. The four measures on children's waiting‐related responses (activity level, amount of button presses per second, observed behavioral and emotional agitations) were positively correlated (*r*s ≥ .21; *p*s ≤ .027). Descriptive statistics of children's waiting‐related responses are shown in Table S1. Factor analyses supported a single latent factor for these four variables which explained 60.8% of their variance (Table S2). This factor was termed *child maladaptive waiting‐related responses*. Parents' use of positive content, positive affect and reciprocity were positively correlated (*r*s ≥ .31; *p*s ≤ .001), while their use of negative content significantly correlated with negative affect (*r* = .70; *p* ≤ .001). Descriptive statistics for parents' responses are shown in Table S3. Factor analysis gave a two‐factor solution explaining 72.1% of their variance. The two factors were termed *parental positive reactions during waiting* and *parental negative reactions during waiting*.

**Table 2:** Intercorrelations between (i) children's waiting‐related responses and (ii) parents' reactions in PC‐DeFT

			1	2	3	4	5	6	7	8
1	Child's waiting‐related responses in PC‐DeFT	Activity level								
2		Amount of button presses	.45**							
3		Behavioral agitation	.54**	.41**						
4		Emotional agitation	.48**	.21	.69**					
5	Parent's reactions in PC‐DeFT	Positive content	.05	.07	.11	.06				
6		Positive affect	−.07	.05	−.06	−.17	.31*			
7		Reciprocity	−.14	−.07	−.18	−.29*	.32**	.67**		
8		Negative content	.52**	.42**	.48**	.48**	−.02	−.05	−.10	
9		Negative affect	.60**	.48**	.49**	.49**	−.01	−.07	−.21	.70**

*p < .01, **p < .001 (adjusted *p* values based on Bonferroni correction).

### Covariates

Child participants' age (both baseline and follow‐up) and IQ were not significantly correlated with teacher‐rated ADHD symptom and delay aversion levels at follow‐up (*r*s ≤ .28, *p*s ≥ .013; Table S4). The ratings did not differ by (i) child's sex (*F*s ≤ 3.55, *p*s ≥ .062), (ii) parent's age (*F*s ≤ 1.34, *p*s ≥ .266), (iii) parent's ethnicity (*F*s ≤ 1.17, *p*s ≥ .313), (iv) parent's educational level (*F*s ≤2.83, *p*s ≥ .042), or (v) household income (*F*s ≤ .43, *p*s ≥ .733; Table S5). These factors were not included in subsequent analyses.

### Baseline association between ADHD behaviors and child's and parent's responses during waiting

Table 3 shows the correlations between children's teacher‐rated ADHD behaviors, their maladaptive waiting‐related responses and parents' reactions during waiting in the PC‐DeFT at baseline. Children's baseline ADHD behaviors as rated by teachers were positively correlated with their maladaptive waiting‐related responses during the PC‐DeFT (*r* = .60, *p* < .001). Parental negative reactions during waiting, but not their positive reactions, were correlated with both their children's baseline ADHD behaviors (*r* = .41, *p* < .001 vs. *r* = −.23, *p* = .017) and maladaptive waiting‐related responses (*r* = .70, *p* < .001 vs. *r* = −.05, *p* = .602). More parental negative reactions during waiting were associated with higher levels of ADHD and more maladaptive waiting‐related responses.

**Table 3:** Correlations between children's teacher‐rated ADHD symptoms, their maladaptive waiting‐related responses and parents' reactions during waiting in PC‐DeFT at baseline (T1)

		1	2	3
Child measures (T1)				
1	ADHD symptoms rating			
2	Maladaptive waiting‐related responses	.60**		
Parent measures (T1)				
3	Negative reactions during waiting	.41**	.70**	
4	Positive reactions during waiting	−.23	−.05	.00

* *p* < .01; ** *p* < .001 (adjusted *p* values based on Bonferroni correction).

### Association between parental reactions in waiting and nonwaiting settings at baseline and children's ADHD symptom and delay aversion levels at follow‐up

Correlational analyses showed that parental negative reactions during waiting on the PC‐DeFT were significantly correlated with children's ADHD behaviors and delay aversion at follow‐up, even after baseline ADHD ratings were controlled for (*r* = .44, *p* < .001 and *r* = .30, *p* = .008, respectively; Table S6). On the other hand, parents' positive reactions during PC‐DeFT, and negative reactions in nonwaiting settings (free play and clean‐up) were not significantly correlated with children's teacher‐rated ADHD behaviors and delay aversion at follow‐up (*r*s ≤ .15, *p*s *≥* .204).

Table 4 shows the regression analyses with children's teacher‐rated ADHD behaviors and delay aversion ratings at follow‐up as outcome variables. Parental negative reactions during waiting in PC‐DeFT was a significant predictor of children's teacher‐rated ADHD and delay aversion ratings at follow‐up (*β*s ≥ .35, *p*s ≤ .008), while parental negative reactions in the two nonwaiting settings were not significant predictors (*β*s ≤ .18, *p*s ≥ .089) (model 1). Parental negative reactions during waiting remained a significant predictor (*β* = .39, *p* < .001 and *β* = .42, *p* = .001 for ADHD and delay aversion ratings at follow‐up, respectively) after children's ADHD behaviors and maladaptive waiting‐related responses at baseline were added as covariates (model 2). Additional analyses were conducted to determine whether the relationships found with the total ADHD score would be different to that for children's inattention and hyperactivity‐impulsivity ratings separately. There was a significant association between parental negative reactions during waiting in the PC‐DeFT and ratings of both inattention and hyperactivity‐impulsivity after controlling for children's baseline ratings (Table S7). These findings suggest that the observed relationships are consistent across different ADHD symptom subdomains.

**Table 4:** Regression models of children's teacher‐rated ADHD symptoms/delay aversion ratings at follow‐up, with (Model 1) parents' waiting‐related reactions at baseline as predictors and (Model 2) parental negative reactions during waiting in PC‐DeFT as predictor, controlling for children's ADHD symptoms and maladaptive waiting‐related responses at baseline

	Predictors (baseline)	Outcome variables at follow‐up			
		Teacher‐rated ADHD symptoms		Teacher‐rated delay aversion	
		*β*	*t*	*β*	*t*
Model 1	Parental positive reactions during waiting in PC‐DeFT	−.13	−1.22	−.03	−0.28
	Parental negative reactions during waiting in PC‐DeFT	.48	4.25**	.35	2.74*
	Parental negative reactions during free play	.18	1.73	.13	1.12
	Parental negative reactions during clean‐up	.18	1.65	.17	1.32
	*R* ^2^	.41		.24	
	*F*	9.92**		4.41*	
Model 2	Parental negative reactions during waiting in PC‐DeFT	.39	3.44**	.42	3.32*
	Children's baseline ADHD symptoms	.45	4.64**	.57	5.37**
	Children's maladaptive waiting‐related responses in PC‐DeFT	.06	.48	−.19	−1.30
	*R* ^2^	.54		.45	
	*F*	28.91**		20.20**	

*p < .01, **p < .001.

### Does delay aversion statistically mediate the association between parental negative reactions during waiting at baseline and ADHD ratings at follow‐up?

**Figure 2:**
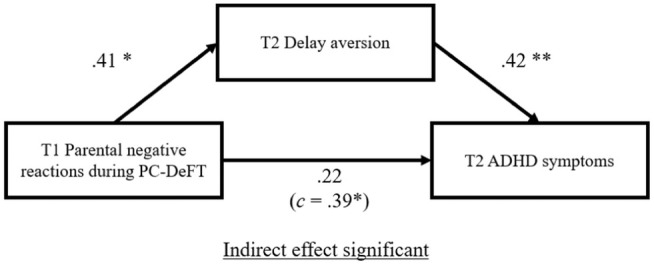
The mediating role of delay aversion in the relationship between parental negative reactions during waiting at baseline (T1) and children's teacher‐rated ADHD symptoms at follow‐up (T2), controlling for baseline ADHD ratings and waiting‐related responses Standardized coefficients shown. * *p* < .01; ** *p* < .001.

With children's baseline ADHD ratings and maladaptive waiting‐related responses controlled for as covariates, PROCESS macro test of mediation showed that the prediction of ADHD behaviors at follow‐up from parental negative reactions during waiting was fully mediated by children's delay aversion, resulting in an insignificant direct effect (Figure 2). The indirect effect was found to be significant (*β* = .17, 95% CI = 0.06, 0.31). On the other hand, both the direct and indirect effects in the relationship between parental negative reactions during nonwaiting settings (free play and clean‐up) and children's ADHD behaviors at follow‐up were not significant (see Figure S1).

### Cross‐cultural comparisons

Table 5 shows the between national‐group differences in child's ratings, task responses, and parents' responses and parental reactions in waiting and nonwaiting settings. There were no significant differences between UK and HK participants on most of the baseline and follow‐up measures. A small group difference was found in children's maladaptive waiting‐related responses, with UK children being more active and agitated during the waiting period in PC‐DeFT than HK children, *F* = 4.09, *p* = .046, $\eta_{p}^{2}$ = .04. UK parents showed higher level of reactions, both positive and negative, during waiting than HK parents, but the differences were not statistically significant (*F*s ≤ .92, *p*s ≥ .339, $\eta_{p}^{2}$s = .01).

**Table 5:** Main effects of national group on all child and parent measures at baseline (T1) and follow‐up (T2)

		UK			HK			Statistical comparison		
		*M*	*SD*	*n*	*M*	*SD*	*n*	*F*	*p*	$\eta_{p}^{2}$
Child measures										
1	Children's maladaptive waiting‐related responses at T1	0.21	1.06	49	−0.19	0.91	54	4.09*	.046	.04
2	Teacher‐rated ADHD symptom at T1	−0.07	1.13	55	0.06	0.86	57	0.46	.498	.00
3	Teacher‐rated ADHD symptom at T2	−0.25	0.94	33	0.17	1.02	47	3.51	.065	.04
4	Teacher‐rated delay aversion at T2	2.13	0.96	33	2.38	0.62	47	1.94	.167	.02
Parent measures (T1)										
5	Parental positive reactions during waiting in PC‐DeFT	0.10	0.97	50	−0.09	1.02	56	0.92	.339	.01
6	Parental negative reactions during waiting in PC‐DeFT	0.08	1.07	50	−0.07	0.93	56	0.63	.430	.01
7	Parental negative reactions during free play	−0.13	0.87	55	0.12	1.10	57	1.71	.194	.02
8	Parental negative reactions during clean‐up	0.08	1.11	47	−0.09	0.85	38	0.60	.441	.01

* *p* < .05.

Further split‐sample correlational analyses showed that patterns of association between parental reactions in waiting and nonwaiting settings and children's levels of teacher‐rated delay aversion and ADHD behaviors at follow‐up were similar in the UK and HK samples. Correlation coefficients between variables in the two samples were compared using Fisher's *Z*‐transformation. *Z*‐tests showed that there were no significant differences between the correlation coefficients in the UK and HK samples (*Z*s ≤ 1.21, *p* ≥ .226; Table S8). PROCESS macro test of moderated mediation analysis (model 59) also demonstrated that mediating role of delay aversion in the relationship between baseline parental negative reactions during waiting and their children's levels of ADHD behaviors was not statistically different for UK and HK participants (Index of moderated mediation = −0.07; 95% CI = −0.22, 0.07).

## Discussion

Existing ADHD models emphasize genetic and biological factors (Banaschewski, Becker, Scherag, Franke, & Coghill, 2010; Faraone & Mick, 2010; Sharma & Couture, 2014; Tarver et al., 2014). Sonuga‐Barke et al. (1992, 2005) proposed a sociomotivational alternative of ADHD development in which hyperactivity, inattention and impulsiveness could be seen as behavioral manifestations of delay aversion, a motivational drive to avoid or escape waiting situations. Further, he argued that delay aversion can be exacerbated in early childhood during negative interactions with significant others in waiting settings which in turn can lead to increases in ADHD behaviors. Here we report the findings from a proof‐of‐concept study designed specifically to test the predictions derived from this hypothesis in a nonclinical sample of preschoolers. There were a number of findings to note.

First, there was a moderate correlation between children's *maladaptive waiting‐related responses* and *parental negative reactions during waiting* on the PC‐DeFT at baseline: the more behaviorally and emotionally agitated a child was in the waiting context, the higher the level of parental negativity. This finding is consistent with the notion that children's reactions to challenging settings (in this case being required to wait) have the power to evoke negative reactions from parents. However, because the data at baseline is cross‐sectional, the possibility that parents' negative reactions also influenced children's response to delay cannot be ruled.

Second, the primary developmental prediction of the delay aversion hypothesis was supported—parental negativity in response to children's waiting‐related difficulties was associated over time, with an exacerbation of ADHD symptom levels. High levels of parental negative reactions during waiting at baseline predicted more ADHD behaviors at follow‐up, even after controlling for baseline ADHD behaviors and children's PC‐DeFT responses.

Third, the longitudinal pathway between parental negative reactions and ADHD behaviors was mediated by children's level of delay aversion. The results therefore are consistent with the delay aversion hypothesis that posits that negative affective states during waiting can develop over time through the pairing of delay experience with the negative affect when children are exposed to parental negative reactions. Another possibility is that observational learning or modeling is contributing to these effects, where children observe and mimic parents' negative emotions during waiting. Future research could explore these different mechanisms using a longitudinal design, exposing children to their parents' waiting performance without direct involvement to examine the relationship between parents' waiting behavior and children's responses in similar situations.

Fourth, the observed associations between parental reactions and children's ADHD were specific to the delay task. Parents' negative reactions in free play and clean‐up tasks were not associated with children's ADHD behaviors or delay aversion longitudinally. Previous studies examining the relationship between parenting practices and ADHD trajectories have not investigated the context specificity of effects, which might account for their generally negative findings (Karreman, van Tuijl, van Aken, & Dekovic, 2006; Mauro & Harris, 2000; Tarver et al., 2014). This aspect of our findings emphasizes the important of taking context into account when examining social environmental impacts on ADHD trajectories. In doing this, it also raises questions about whether parental responses in other settings may have similar effects to those in the delay setting (i.e. setting involving other emotionally challenging events). It also highlights issues around assessment and whether ADHD rating scales should take more account of context—with ADHD being more highly rated in delay than nondelay settings.

Fifth, no significant differences were found between UK and HK children and parents across various dimensions. The effect of parental negative reactions during waiting on children's ADHD behaviors via the development of delay aversion was also consistent across cultures. This highlights the relative importance of proximal social processes on ADHD symptom trajectories over more distal cultural factors.

The study's strengths included its longitudinal design, a substantial sample across two cultures, the use of multiple tools and informants across two time points to minimize shared‐method variance, and the use of observation and coding of parental reactions in both waiting and nonwaiting settings, allowing for contextual effects on children's delay aversion and ADHD behaviors to be tested. However, there were also limitations. First, the originally planned follow‐up which involved children and parents participating in all tasks at both time points was not implemented due to COVID‐19 lockdown. Nonetheless, data from questionnaires at follow‐up still allowed us to test the core hypothesis. Second, we acknowledge that having to collect follow‐up data during the pandemic may have affected parents' stress levels and, consequently, their ratings of children's behaviors. Third, although we observed and coded parents' interactions with their children in both waiting and nonwaiting situations to examine the context specificity of the relationship between parental responses and children's ADHD symptoms, the nonwaiting situations in our study did not appear to elicit similar levels of negative affect as the waiting situations. It would be valuable to contrast parent–child interactions during waiting tasks with other potentially equally aversive situations, such as difficult tasks, games involving frequent losses, or situations requiring parents to set limits or control their children's behaviors, to test whether the associations between parental reactions and children's ADHD behaviors are specifically tied to delay situations rather than other aversive situations. Fourth, the number of delay trials and the duration of unexpected delay in PC‐DeFT was relatively short – designed to be age‐appropriate for preschoolers. Future research could explore a wider range of waiting durations. Fifth, the study included only few cases in the clinical range and so little can be said about whether parental reactions to children's responses can contribute to clinical expressions of ADHD. Sixth, there was only two assessment points and so we were unable to measure delay aversion at a point intermediate between baseline and outcome measures, which limited the interpretation of the mediation effect.

These findings have several potential clinical implications. Interventions targeting parent–child interaction in delay‐rich waiting settings may be valuable, providing strategies for both parents and children to navigate delay more effectively. Kopp's (1989) research on self‐regulation suggested that young children required external regulation from parents before intrinsic self‐regulatory abilities fully develop. However, some studies indicated that, without guidance, parents may lack sufficient knowledge of effective waiting strategies: they often encouraged children to focus on waiting or the delayed reward, which were found to be associated with a decrease in children's ability to wait (Hom & Knight, 1996; Mauro & Harris, 2000; Mischel & Baker, 1975; Mischel & Ebbesen, 1970). In contrast, children with parents who explained, helped them cope with waiting‐related stress and frustration, or taught distraction strategies performed better in delay‐of‐gratification tasks (LeCuyer‐Maus & Houck, 2002; Peake, Mischel, & Hebl, 2002). To support children's development of the ability to cope with delay, it is likely to be important for parents themselves to develop effective self‐regulation skills, where these are lacking. Negative parental reactions in response to their children's behavior may stem from their own struggles with delay aversion or their difficulty managing stress and frustration triggered by their child's behaviors. Also, parents equipped with regulatory skills can model important characteristics such as calmness during interactions with their children and also reinforce children's successful waiting behaviors so waiting becomes associated with a more peaceful state and neutral or positive affect. The cross‐cultural invariance found in the delay aversion model suggested that these early intervention efforts could be useful in different cultures.

In summary, we provide evidence that parental negative reactions to preschool children's maladaptive delay‐related responses are associated with ADHD longitudinal behavioral trajectories in a nonclinical sample over a relatively short period of time.

## Ethical considerations

The authors assert that all procedures contributing to this work comply with the ethical standards of the relevant national and institutional committees on human experimentation and with the Helsinki Declaration of 1975, as revised in 2008. This study was reviewed and approved by Research Ethics Committee of the University of Hong Kong [EA1812027] and King's College London [HR‐18/19‐8506]. Informed consent was obtained from all individual participants included in the study.

**Key points**

- Prior studies have largely failed to demonstrate the power of the postnatal social environment to determine trajectories of ADHD behaviors.
- Sonuga‐Barke hypothesized that parent's negative responses specifically in the context of children's maladaptive waiting responses will increase levels of delay aversion and so ADHD behaviors.
- We tested this in a longitudinal study using a new delay frustration task.
- Baseline levels of parent's negative reactions to children's maladaptive responses to the delay frustration task predicted elevated levels of ADHD behaviors at follow‐up (12 to 18 months later), after controlling for baseline ADHD behaviors—effects mediated by delay aversion.
- The effects were not seen for parental responses in nondelay settings.
- The same pattern of results was seen in samples from the UK and Hong Kong.

## Supporting information


**Appendix S1.** Summary of the PARCHISY codes applied in this study.
**Figure S1.** The mediating role of delay aversion in the relationship between parental negative reactions during free play (above)/clean‐up (below) at baseline (T1) and children's teacher‐rated ADHD symptoms at follow‐up (T2), controlling for baseline ADHD ratings and waiting‐related responses.
**Table S1.** Descriptive statistics of children's responses in PC‐DeFT, free play and clean‐up.
**Table S2.** Exploratory factor analysis for child's waiting‐related responses in PC‐DeFT.
**Table S3.** Descriptive statistics of PARCHISY codes used in the three parent–child interaction tasks.
**Table S4.** Correlations between children's IQ, age, ADHD and delay aversion ratings at baseline (T1) and follow‐up (T2).
**Table S5.** Child's sex, parent characteristics and household background differences in ADHD and delay aversion ratings at baseline (T1) and follow‐up (T2).
**Table S6.** Partial correlation between parental reactions during waiting and nonwaiting settings and the children's teacher‐rated ADHD symptoms and delay aversion at follow‐up (T2), controlling for ADHD ratings at baseline (T1).
**Table S7.** Regression models of children's teacher‐rated ADHD symptoms at follow‐up.
**Table S8.** The partial correlation between parental reactions during waiting and nonwaiting settings and the children's teacher‐rated ADHD symptoms and delay aversion at follow‐up between UK and HK participants, controlling for baseline ratings.

## Data Availability

The data that support the findings of this study and other study materials are available on request from the corresponding author (ESB).
