# Supplementary material for: Are ADHD trajectories shaped by the social environment? A longitudinal study of maternal influences on the preschool origins of delay aversion
Source: J Child Psychol Psychiatry. 2024 Dec 22;66(6):892–905. doi: 10.1111/jcpp.14103 (PMC12062859; doi:10.1111/jcpp.14103)
Supplement: Supplementary file 1 — Appendix S1. Summary of the PARCHISY codes applied in this study. Figure S1. The mediating role of delay aversion in the relationship between parental negative reactions during free play (above)/clean‐up (below) at baseline (T1) and children's teacher‐rated ADHD symptoms at follow‐up (T2), controlling for baseline ADHD ratings and waiting‐related responses. Table S1. Descriptive statistics of children's responses in PC‐DeFT, free play and clean‐up. Table S2. Exploratory factor analysis for child's waiting‐related responses in PC‐DeFT. Table S3. Descriptive statistics of PARCHISY codes used in the three parent–child interaction tasks. Table S4. Correlations between children's IQ, age, ADHD and delay aversion ratings at baseline (T1) and follow‐up (T2). Table S5. Child's sex, parent characteristics and household background differences in ADHD and delay aversion ratings at baseline (T1) and follow‐up (T2). Table S6. Partial correlation between parental reactions during waiting and nonwaiting settings and the children's teacher‐rated ADHD symptoms and delay aversion at follow‐up (T2), controlling for ADHD ratings at baseline (T1). Table S7. Regression models of children's teacher‐rated ADHD symptoms at follow‐up. Table S8. The partial correlation between parental reactions during waiting and nonwaiting settings and the children's teacher‐rated ADHD symptoms and delay aversion at follow‐up between UK and HK participants, controlling for baseline ratings. [file JCPP-66-892-s001.docx]

**Supporting document**

**Appendix S1. Summary of the PARCHISY codes applied in this study**

| (A) Positive content/control: use of praise, explanation, and open-ended questions | |
| --- | --- |
|  | - 1. No positive control shown   2. One or two instances of positive control   3. A few/several instances of positive control; reliance on explicit directions (“up, down, stop”)   4. Moderate amounts of positive control shown; reliance on explicit directions with at least one instance of praise, explanation, or questioning   5. Two or more instances of explanation, questioning, or praise, with some explicit directions   6. Substantial use of explanation, questioning, and praise, and few explicit directions; only one or two instances of non-positive control shown   7. Exclusive use of explanation, questioning, and praise |
| (B) Positive affect/ warmth: smiling, laughing | |
|  | 1. No positive affect displayed 2. One or two instances of positive affect 3. A few/several instances of positive affect 4. Moderate amounts of positive affect - smiling, laughing for about half of interaction 5. Positive affect for more than half of interaction 6. Substantial amounts of positive affect; only one or two instances of non-positive affect 7. Constant positive affect - smiling and laughing throughout task |
| (C) Reciprocity: shared positive affect, eye contact, a “turn taking” quality of interaction | |
|  | 1. No evidence of reciprocity 2. One or two instances of reciprocity - either shared affect or eye contact 3. A few/several instances of reciprocity (either shared affect or eye contact) 4. Moderate levels of reciprocity; evidence of both shared affect and eye contact; some evidence of “conversation-like” interaction 5. Clear evidence of reciprocity; one or two episodes of intense shared positive affect coupled with eye contact that is sustained for several “turns” between parent and child 6. Substantial reciprocity involving numerous episodes of intense shared positive affect coupled with eye contact that is sustained for several “turns”; only one or two instances of non-reciprocity 7. Highly integrated and reciprocal - constant shared positive affect and eye contact that never loses “turn taking” quality |
| (D) Negative content/control: use of physical control of child’s hand/arm/body, use of criticism | |
|  | 1. No negative control shown 2. One or two instances of negative control 3. A few/several instances of negative control 4. Moderate amounts of negative control: reliance on critical comments (“no, don’t do that”) and/or manipulation of dials 5. Negative control used for more than half the interaction 6. Substantial use of criticism, and physically “taking over” task; only a few instances of non-negative control shown 7. Exclusive use of criticism (can include shaming) and physical control of dials and/or child’s hand/arm/body; may include instances of corporal punishment |
| (E) Negative affect/ rejection: frowning, cold/harsh voice | |
|  | 1. No negative affect displayed 2. One or two instances of negative affect 3. A few/several instances of negative affect 4. Moderate amounts of negative affect - frowning, stern looking, harsh/cold voice for about half of interaction 5. Negative affect for more than half of interaction 6. Substantial amounts of negative affect; only one or two instances of non-negative affect 7. Constant negative affect - always scowling/frowning, voice always in harsh tones |

**Table S1. Descriptive statistics of children’s responses in PC-DeFT, free play and clean-up**

|  |  | **UK** | | |  | **HK** | | |  | Statistical comparison | | |
| --- | --- | --- | --- | --- | --- | --- | --- | --- | --- | --- | --- | --- |
|  |  | *M* | SD | *n* |  | *M* | SD | *n* |  | *F* | *p* | *η_p_*^2^ |
| Child’s waiting-related responses in PC-DeFT | |  |  |  |  |  |  |  |  |  |  |  |
| 1 | Activity level | 179.87 | 64.26 | 49 |  | 153.33 | 66.60 | 54 |  | 4.22 | .043 | .04 |
| 2 | Amount of button presses | 3.60 | 3.87 | 50 |  | 3.58 | 3.20 | 56 |  | .00 | .979 | .00 |
| 3 | Behavioural agitation | 2.79 | .91 | 50 |  | 2.46 | .95 | 56 |  | 3.28 | .073 | .03 |
| 4 | Emotional agitation | 1.73 | .67 | 50 |  | 1.46 | .58 | 56 |  | 5.17 | .025 | .05 |
|  |  |  |  |  |  |  |  |  |  |  |  |  |
| Child’s responses during free play | |  |  |  |  |  |  |  |  |  |  |  |
| 1 | Activity level | 203.22 | 43.45 | 52 |  | 196.96 | 46.15 | 55 |  | .52 | .47 | .01 |
| 2 | Emotional agitation | 1.00 | .00 | 55 |  | 1.04 | .27 | 57 |  | .97 | .33 | .01 |
|  |  |  |  |  |  |  |  |  |  |  |  |  |
| Child’s responses during clean-up | |  |  |  |  |  |  |  |  |  |  |  |
| 1 | Activity level | 224.22 | 58.73 | 46 |  | 217.30 | 61.71 | 55 |  | .33 | .57 | .00 |
| 2 | Emotional agitation | 1.19 | .45 | 47 |  | 1.13 | .34 | 38 |  | .46 | .50 | .01 |

Table S2. Exploratory factor analysis for child’s waiting-related responses in PC-DeFT

| **Measure** | **Factor loadings** |
| --- | --- |
| Activity level | .794 |
| Amount of button presses | .637 |
| Behavioural agitation | .878 |
| Emotional agitation | .790 |
|  |  |
| **Eigenvalue** | 2.43 |
| **Variance (%)** | 60.82 |
| KMO = .67; Bartlett’s Test of Sphericity = 136.67 (*df* = 6, *sig* < .001). | |

**Table S3. Descriptive statistics of PARCHISY codes used in the three parent-child interaction tasks**

| Variables | Free Play | | Clean-up | | PC-DeFT | |
| --- | --- | --- | --- | --- | --- | --- |
|  | Mean | SD | Mean | SD | Mean | SD |
| (A) Positive content | 4.68 | 1.58 | 3.82 | 1.58 | 2.67 | 1.82 |
| (B) Positive affect | 2.85 | 1.06 | 1.75 | .65 | 2.42 | .74 |
| (C) Reciprocity | 2.74 | .97 | 1.58 | .71 | 1.96 | .89 |
| (D) Negative content | 1.21 | .47 | 1.28 | .53 | 1.54 | .76 |
| (E) Negative affect | 1.06 | .34 | 1.16 | .40 | 1.30 | .59 |

**Table S4. Correlations between children’s IQ, age, ADHD and delay aversion ratings at baseline (T1) and follow-up (T2)**

|  |  |  | **IQ** | **Age at T1** | **Age at T2** |
| --- | --- | --- | --- | --- | --- |
| 1 | ADHD symptom ratings at T1 | | -.24 | -.16 | -.13 |
| 2 | ADHD symptom ratings at T2 | | -.20 | -.24 | -.22 |
| 3 | Delay aversion ratings at T2 | | -.14 | -.28 | -.28 |

Note: * *p* < .01; ** *p* < .001 (adjusted *p* values based on Bonferroni correction).

**Table S5. Child's sex, parent characteristics and household background differences in ADHD and delay aversion ratings at baseline (T1) and follow-up (T2)**

|  |  | **Difference between** | | | | |
| --- | --- | --- | --- | --- | --- | --- |
|  |  | **Child’s sex** | **Parent’s age group** | **Parent’s ethnic group** | **Parent’s education level** | **Household income group** |
| 1 | ADHD symptom ratings at T1 | *F* ^a^ = 3.55, *p* = .062 | *F* ^b^ = 1.34, *p* = .266 | *F* ^b^ = 1.17, *p* = .313 | *F* ^c^ = 2.83, *p* = .042 | *F* ^c^ = .43, *p* = .733. |
| 2 | ADHD symptom ratings at T2 | *F* ^d^ = 2.75, *p* = .101 | *F* ^e^ = .45, *p* = .642 | *F* ^e^ = .65, *p* = .523 | *F* ^f^ = 1.01, *p* = .393 | *F* ^f^ = .11, *p* = .956. |
| 3 | Delay aversion ratings at T2 | *F* ^d^ = 3.01, *p* = .087 | *F* ^e^ = .96, *p* = .387 | *F* ^e^ = .44, *p* = .643 | *F* ^f^ = 2.26, *p* = .088 | *F* ^f^ = .31, *p* = .820. |

Note. The *df* of variables for *t* statistics are *a* = (1, 110); *b* = (2, 109); *c* = (3, 108); *d* = (1, 78); *e* = (2, 77); *f* = (3, 76) respectively.

**Table S6. Partial correlation between parental reactions during waiting and non-waiting settings and the children’s teacher-rated ADHD symptoms and delay aversion at follow-up (T2), controlling for ADHD ratings at baseline (T1)**

| Baseline measures | | | Outcome variables at follow-up | | |
| --- | --- | --- | --- | --- | --- |
|  |  |  | ADHD symptoms |  | Delay aversion |
| 1 | Parental positive reactions during | Waiting in PC-DeFT | -.03 |  | .03 |
| 2 | Parental negative reactions during | Waiting in PC-DeFT | .44** |  | .30** |
| 3 |  | Free play | .15 |  | .09 |
| 4 |  | Clean-up | .15 |  | .09 |

Note: Controlling for baseline data. * *p* < .01; ** *p* < .001 (adjusted *p* values based on Bonferroni correction).

**Table S7. Regression models of children’s teacher-rated ADHD symptoms at follow-up**

|  |  | Teacher-rated ADHD symptoms at follow-up | | | | | |
| --- | --- | --- | --- | --- | --- | --- | --- |
|  |  | Inattention subscale | | Hyperactivity- Impulsivity subscale | | Total scale | |
|  | Predictors (baseline) | *β* | *t* | *β* | *T* | *β* | *t* |
| Model 1 | Parental positive reactions during waiting in PC-DeFT | -.12 | -1.05 | -.12 | -1.12 | -.13 | -1.22 |
|  | Parental negative reactions during waiting in PC-DeFT | .42 | 3.52** | .45 | 3.93** | .48 | 4.25** |
|  | Parental negative reactions during free play | .11 | .97 | .22 | 2.07 | .18 | 1.73 |
|  | Parental negative reactions during clean-up | .21 | 1.74 | .13 | 1.17 | .18 | 1.65 |
|  | *R^2^* | .33 | | .38 | | .41 | |
|  | *F* | 6.89** | | 8.67** | | 9.92** | |
|  |  | *β* | *t* | *β* | *T* | *β* | *t* |
| Model 2 | Parental negative reactions during waiting | .36 | 2.75* | .37 | 3.33* | .39 | 3.44** |
|  | Children’s baseline ADHD symptoms | .36 | 3.23* | .48 | 5.05** | .45 | 4.64** |
|  | Children’s maladaptive waiting-related responses in PC-DeFT | .05 | .31 | .07 | .54 | .06 | .48 |
|  | *R^2^* | .39 | | .56 | | .54 | |
|  | *F* | 15.68** | | 31.28** | | 28.91** | |

Note. * *p* < .01; ** *p* < .001.

**Table S8. The partial correlation between parental reactions during waiting and non-waiting settings and the children’s teacher-rated ADHD symptoms and delay aversion at follow-up between UK and HK participants, controlling for baseline ratings**

|  |  | Outcome variables at follow-up | | | | |
| --- | --- | --- | --- | --- | --- | --- |
|  |  | ADHD symptoms | |  | Delay aversion | |
| Baseline measures | | UK | HK |  | UK | HK |
| 1 | Parental positive reactions during waiting in PC-DeFT | -.13 | -.02 |  | .09 | -.01 |
| 2 | Parental negative reactions during waiting in PC-DeFT | .12 | .40* |  | .29 | .16 |
| 3 | Parental negative reactions during free play | .26 | -.05 |  | .04 | .07 |
| 4 | Parental negative reactions during clean-up | -.02 | .19 |  | -.08 | .22 |

Note: Controlling for T1 children’s ADHD symptom ratings and maladaptive waiting-related responses. * *p* < .01; ** *p* < .001 (adjusted *p* values based on Bonferroni correction).


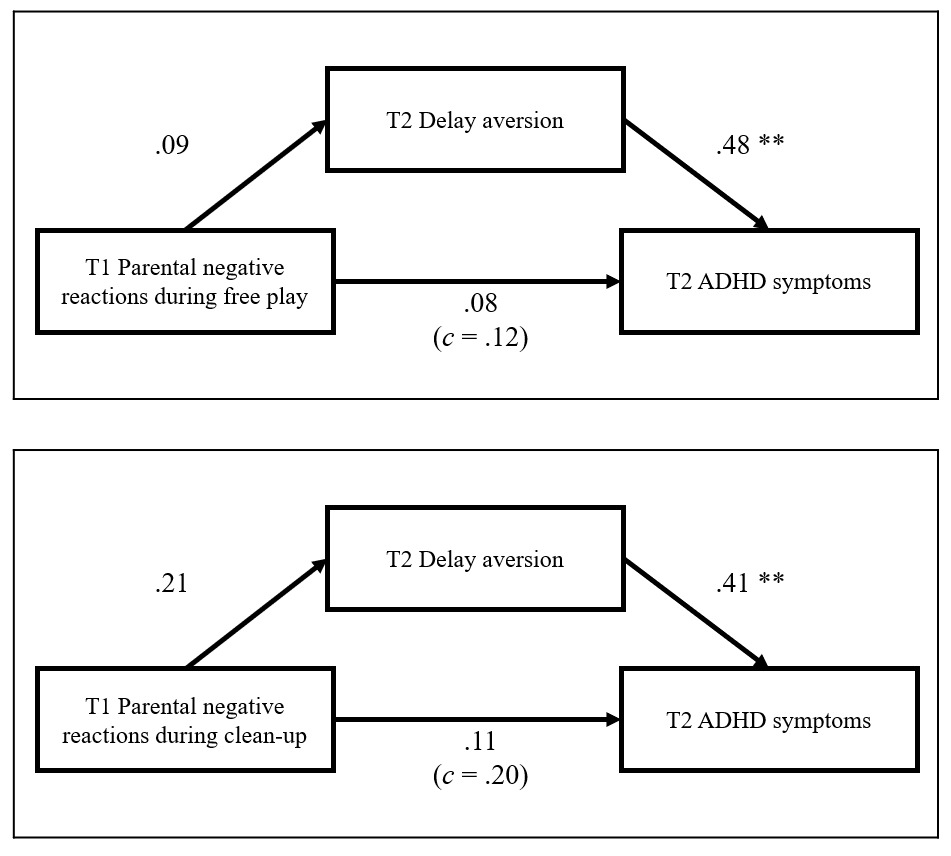


Figure S1. The mediating role of delay aversion in the relationship between parental negative reactions during free play (above)/ clean-up (below) at baseline (T1) and children’s teacher-rated ADHD symptoms at follow-up (T2), controlling for baseline ADHD ratings and waiting-related responses

Note. Standardized coefficients shown. * *p* < .01; ** *p* < .001
